# Supplementary figures and images for: Genome-Wide Identification and an Evolution Analysis of Tonoplast Monosaccharide Transporter (TMT) Genes in Seven Gramineae Crops and Their Expression Profiling in Rice
Source: Genes (Basel). 2023 May 24;14(6):1140. doi: 10.3390/genes14061140 (PMC10297915; doi:10.3390/genes14061140)

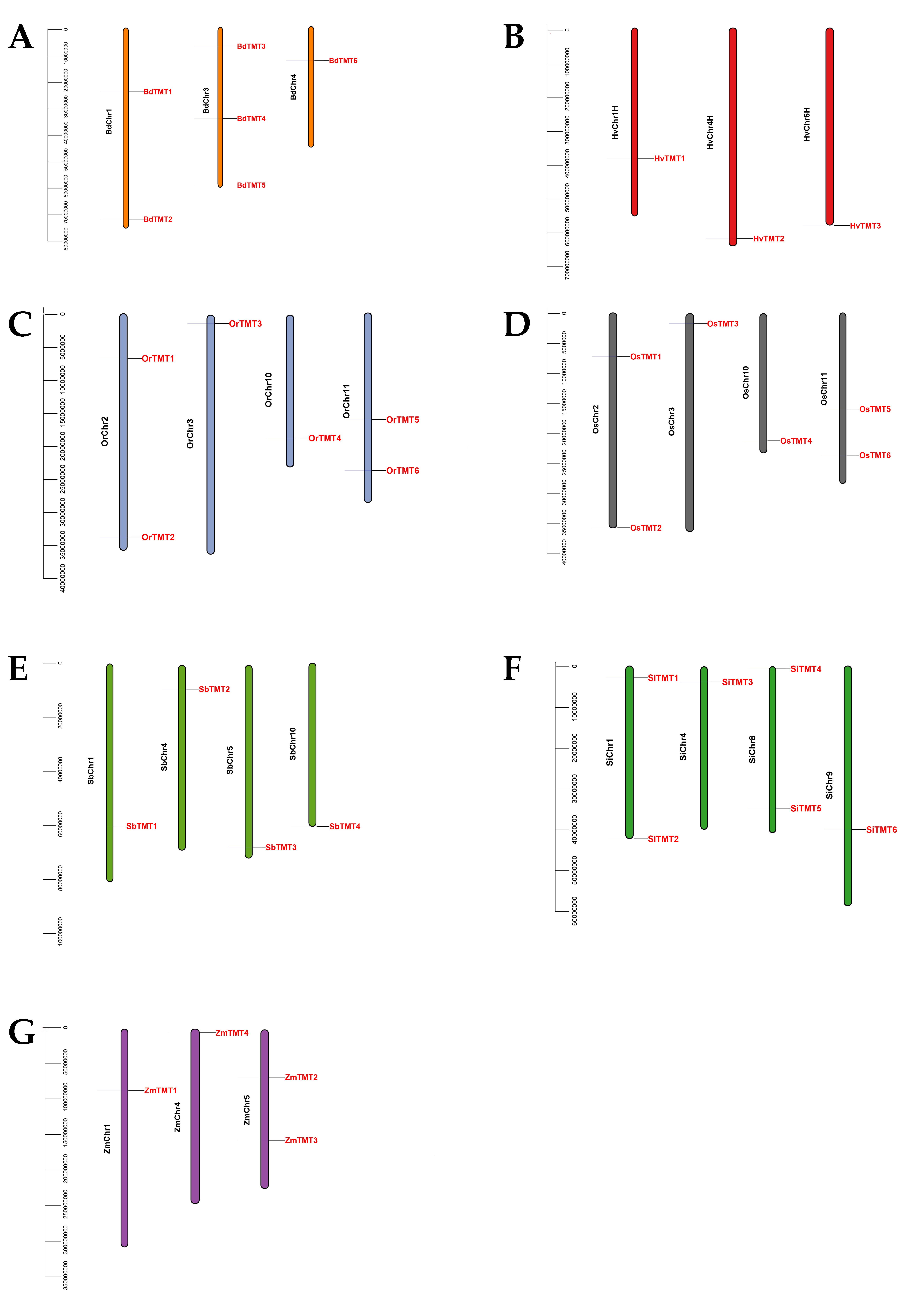

Supplement: Supplementary file 1 [file genes-14-01140-s001.zip › Figure S1.png]
